# Critical appraisal of guidelines for coronary artery disease on dual antiplatelet therapy: More consensus than controversies

**DOI:** 10.1002/clc.23275

**Published:** 2019-10-14

**Authors:** Shaozhao Zhang, Huimin Zhou, Xiaodong Zhuang, Daya Yang, Xiuting Sun, Xiangbin Zhong, Xiaoyu Lin, Xun Hu, Yiquan Huang, Xinxue Liao, Zhimin Du

**Affiliations:** ^1^ Cardiology Department First Affiliated Hospital of Sun Yat‐sen University Guangzhou China; ^2^ NHC Key Laboratory of Assisted Circulation Sun Yat‐sen University Guangzhou China; ^3^ Department of Anesthesiology The Third Affiliated Hospital of Sun Yat‐sen University Guangzhou China

**Keywords:** AGREE II, coronary artery disease, dual antiplatelet therapy, guideline

## Abstract

**Background:**

Dual antiplatelet therapy (DAPT) in the form of aspirin plus a P_2_Y_12_ inhibitor, when indicated, is one of the key treatments in coronary artery disease (CAD). Many recommendations on DAPT in patients with CAD based on current guidelines are largely inconsistent. In our current study, we aimed at systematically reviewing DAPT‐relevant clinical practice guidelines, and highlighting their commonalities and differences for better informed decision‐making.

**Methods:**

Contemporary guidelines in English were searched in MEDLINE, Embase and websites of guideline organizations and professional societies. Guidelines with recommendations on DAPT for CAD patients were included. Guideline quality was appraised with the 6‐domain Appraisal of Guidelines for Research and Evaluation II (AGREE II) instrument. The reporting of conflicts of interest (COI) was assessed individually with supplementary items from the RIGHT (Reporting Item for Practice Guidelines in Healthcare) checklist. Meanwhile, extraction of recommendations was performed.

**Results:**

A total of 18 guidelines fulfilled our inclusion criteria. Most of them were graded with relatively good scores averaging from 42% to 74%. Domains for lower scores were in “stakeholder involvement” and “application.” The reporting of COI was satisfactory. For the recommendations on DAPT, most guidelines with high AGREE II scores included consistent recommendations on the timing and P_2_Y_12_ inhibitor selection. Nonetheless, conflicts still exist on the duration of DAPT.

**Conclusions:**

Quality of guidelines for DAPT in CAD was relatively high, though defects existed in “Applicability” and “Stakeholder Involvement.” As these guidelines developed, DAPT recommendations gradually converged on a consensus. Clinical decision should be made on an individual basis.

## 1 INTRODUCTION

Coronary artery disease (CAD) is the leading cause of morbidity and mortality globally. The high burden of disease and the costs seriously affect individuals, families, and society at large.^1^ Dual antiplatelet therapy (DAPT) in the form of aspirin plus a P_2_Y_12_ inhibitor, when indicated, is one of the key treatments in CAD. For example, it is the secondary prevention for patients after acute coronary syndrome (ACS) and prevents stent thrombosis in patients with recent stent implantation.^2^ In the meantime, clinicians usually rely on evidence‐based clinical guidelines to make decision. As a result, several international organizations have published clinical guidelines (CPGs) with recommendations of DAPT for patients with CAD.

There were more than 10 DAPT‐relevant CPGs published in the past two decades. Though most of them claimed to be evidence‐based, we found a considerable amount of differences in their recommendations, which may be confusing for clinicians. Since the quality of these guidelines remained unclear, critical appraisal of these guidelines is important. Several methodologies have been developed for the assessment of CPGs.^3^ Among these, the Appraisal of Guidelines for Research and Evaluation (AGREE) instrument was widely accepted and also endorsed by the WHO,^4^ which was then updated to AGREE II.^5^ Systematic review of CPGs using AGREE (AGREE II) was documented to be of tremendous value.^6^

## 2 METHODS

### 2.1 Data sources and searches

A systematic literature search for existing guidelines was performed in MEDLINE and Embase from 1 January 1999 to 1 September 2019, using keywords of “coronary artery disease,” “stable coronary artery disease,” “angina,” “acute coronary syndrome” and “guideline.” Additional guidelines were obtained by reviewing websites of guideline organizations and professional societies (see Table S1).

### 2.2 Inclusion and exclusion criteria

Criteria to select guidelines were as follows: (a) articles meeting the Institute of Medicine definition for clinical practice guidelines^7^: “systematically developed statements to assist practitioner and patient decisions about appropriate healthcare for specific clinical circumstance”; (b) target groups included patients with CAD; (c) contained recommendations on DAPT for CAD patients, with the exception of coronary bypass grafting as its strategy is summarily different; (d) the latest available version; (e) published in English. Titles and abstracts were reviewed independently by two reviewers (Z.S.Z and Z.H.M). Decision to preclude any article in the final analysis was reached by both reviewers. Disagreements between two reviewers were discussed and resolved by consensus. The final selection of articles was checked by a third reviewer (S.X.T).

### 2.3 Quality appraisal of guidelines

We assessed the quality of each included guideline using the standardized Appraisal of Guidelines Research and Evaluation instruction II (AGREE II),^5^ which consists of 23 items in six domains: (a) scope and purpose; (b) stakeholder involvement; (c) rigor of development; (d) clarity of presentation; (e) applicability; (f) editorial independence. Each domain was independently rated by two reviewers (Z.S.Z and Z.H.M) who were blinded to each other's ratings. Each item within an individual domain was rated from 1 (strongly disagree) to 7 (strongly agree). For each reviewer, AGREE II scores of each domain were calculated as a percentage using the sum of all items and the maximum possible score. Agreement between reviewers on AGREE II scores was assessed using the intraclass correlation coefficient by two‐way random model with the type of absolute agreement. The final scores of each domain was calculated by combining the scores of each reviewer based on the recommended formula: (*S*_ob_ − *S*_minp_)/(*S*_maxp_ − *S*_minp_), where *S*_ob_ is the obtained score, *S*_minp_ is the minimum possible score, and *S*_maxp_ is the maximum possible score. A guideline was “strongly recommended for use in practice” if most domains (four or more) scored above 60%. A guideline was “recommended for use with some modification” if most domains scored between 30% and 60%. “Not recommended for use in practice” implied that most of the domains of the guideline were scored as approximately or below 30%.^8^, ^9^

In addition, we assessed the reporting of conflicts of interest (COI). Except for the two items in domain 6 of AGREE II, another four items from the RIGHT (Reporting Item for Practice Guidelines in Healthcare) checklist^10^, ^11^ were also appraised by one reviewer (Z.S.Z) and checked by a second reviewer (Z.H.M): (a) specific sources of funding for all stages of guideline development; (b) the role of the funder(s) in guideline development, dissemination, and implementation; (c) the types of COI (financial and nonfinancial) that were relevant to the guideline; (d) how COI were evaluated and managed and how users of the guideline can access the declarations of interests. Besides, the proportion of guideline panel member‐industry relationships was also calculated.^6^ Discrepancies were resolved by discussion.

### 2.4 Data extraction and analysis

All recommendations about DAPT in CAD (with the exception of CABG) from each included guideline were extracted by one reviewer (Z.S.Z). A second reviewer (Z.H.M) checked the result for completeness and accuracy. Discrepancies were resolved by consensus. A table was constructed for comparison of the recommendations. Recommendations were categorized into strategies for patients with percutaneous coronary intervention (PCI), medical therapy or fibrinolytic therapy. Each recommendation was categorized into DAPT initial timing, P_2_Y_12_ inhibitor selection and DAPT duration.

We examined the correlation between the proportion of guideline panel members who reported relationships with industry and the AGREE II score with guideline. The guidelines (2012 JCS and 2018 TSC) did not have explicit statement on COI of panel members was excluded from the analyses. All analyses were performed using SPSS 21.0 and *P* < .05 was considered statistically significant.

## 3 RESULTS

### 3.1 Selected guidelines

**Figure 1:**
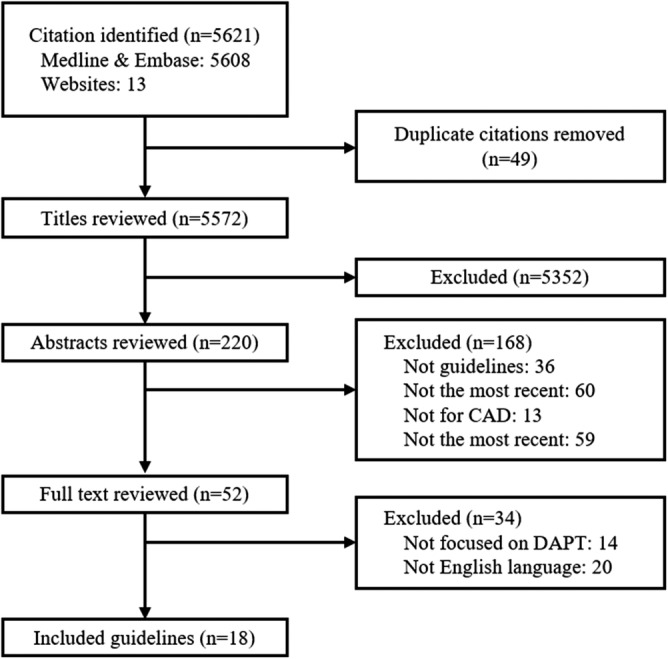
Guidelines search and selection

A total of 18 guidelines from seven organizations were identified (Figure 1). Table 1 summarizes the characteristics of the included guidelines, with COI and the average AGREE II scores. Five guidelines were from European continent,^1^, ^12^, ^13^, ^14^, ^15^ two from the United Kingdom,^16^, ^17^ six from the United States,^18^, ^19^, ^20^, ^21^, ^22^, ^23^ one from Canada,^24^ one from Australia and New Zealand,^25^ two from Japan,^26^, ^27^ and one from Taiwan.^28^

**Table 1:** Characteristics of 18 guidelines on DAPT for coronary artery disease

Guidelines identifier, year	Organization(s) responsible for guidelines development	Region	Target population	Focus on DAPT	Average AGREE score, %	Conflicts of interest	Proportion of pane members with reported industry relationship
ESC, 2019^12^	European Society of Cardiology	Europe	CCS	No	69	aSCI, aSCIR, DSFS, DTCO, DEMC, DADI	22/25
ESC, 2018^13^	European Society of Cardiology	Europe	CAD	No	68	aSCI, aSCIR, DSFS, DTCO, DEMC, DADI	21/22
ESC1, 2017^14^	European Society of Cardiology	Europe	CAD	Yes	69	aSCI, aSCIR, DSFS, DTCO, DEMC, DADI	13/18
ESC2, 2017^1^	European Society of Cardiology	Europe	STE‐ACS	No	66	aSCI, aSCIR, DSFS, DTCO, DEMC, DADI	15/18
ESC, 2015^15^	European Society of Cardiology	Europe	NSTE‐ACS	No	68	aSCI, aSCIR, DSFS, DTCO, DEMC, DADI	17/19
NICE1, 2013^16^	National Institute for Health and Care Excellence	United Kingdom	STE‐ACS	No	70	aSCI, DSFS, DTCO, DEMC, DADI	16/25
NICE2, 2013^17^	National Institute for Health and Care Excellence	United Kingdom	ACS	No	74	aSCI, DADI	8/25
AHA/ACC, 2016^18^	American Heart Association/American College of Cardiology	United States	CAD	Yes	66	aSCI, aSCIR, DIR, DSFS, DTCO, DEMC, DADI	7/17
AHA/ACC, 2014^19^	American Heart Association/American College of Cardiology	United States	NSTE‐ACS	No	70	aSCI, aSCIR, DIR, DSFS, DTCO, DEMC, DADI	6/17
AHA/ACCF, 2013^20^	American Heart Association/American College of Cardiology Foundation	United States	STE‐ACS	No	72	aSCI, aSCIR, DIR, DSFS, DTCO, DEMC, DADI	12/23
AHA/ACCF1, 2012^21^	American Heart Association/American College of Cardiology Foundation	United States	UA and NSTE‐ACS	No	62	aSCI, aSCIR, DIR, DSFS, DTCO, DEMC, DADI	7/15
AHA/ACCF2, 2012^22^	American Heart Association/American College of Cardiology Foundation	United States	SCAD	No	64	aSCI, aSCIR, DIR, DSFS, DTCO, DEMC, DADI	16/26
AHA/ACCF, 2011^23^	American Heart Association/American College of Cardiology Foundation	United States	CAD	No	62	aSCI, aSCIR, DIR, DSFS, DTCO, DEMC, DADI	11/18
CCS, 2018^24^	Canadian Cardiovascular Society	Canada	CAD	No	58	aSCI, aSCIR, DSFS, DTCO, DADI	14/22
NHFA/CSANZ, 2016^25^	National Heart Foundation of Australia/Cardiac Society of Australia and New Zealand	Australia and New Zealand	ACS	No	54	aSCI, DSFS, DTCO, DEMC, DADI	7/10
JCS, 2012^26^	Japanese Circulation Society	Japan	SCAD	No	42	—	—
JCS, 2018^27^	Japanese Circulation Society	Japan	ACS	No	54	aSCI, DTCO, DADI	48/48
TSC, 2018^28^	Taiwan Society of Cardiology	Taiwan, China	NSTE‐ACS	No	50	DIR, DSFS	—

Abbreviations: ACS, acute coronary syndrome; CAD, coronary artery disease; CCS, chronic coronary syndrome; DADI, disclosure of how to access the declarations of interests; DAPT, dual antiplatelet therapy; DEMC, disclosure of the evaluation and management of the COI; DIR, disclosure of the identities of peer reviews; DSFS, disclosure of the specific sources of funding for all stages of guideline development; DTCO, disclosure the types of COI (financial and nonfinancial) that are relevant to the guidelines; NSTE‐ACS, non‐ST‐elevation acute coronary syndrome; SCAD, stable coronary artery disease; SCI, statement about conflicts of interest of panel members present; SCIR, statement about conflicts of interest of external peer reviews present; STE‐ACS, ST‐elevation acute coronary syndrome; UA, unstable angina.

a Relationship with industry reported by at least one person.

### 3.2 Guideline appraisal by AGREE II

**Figure 2:**
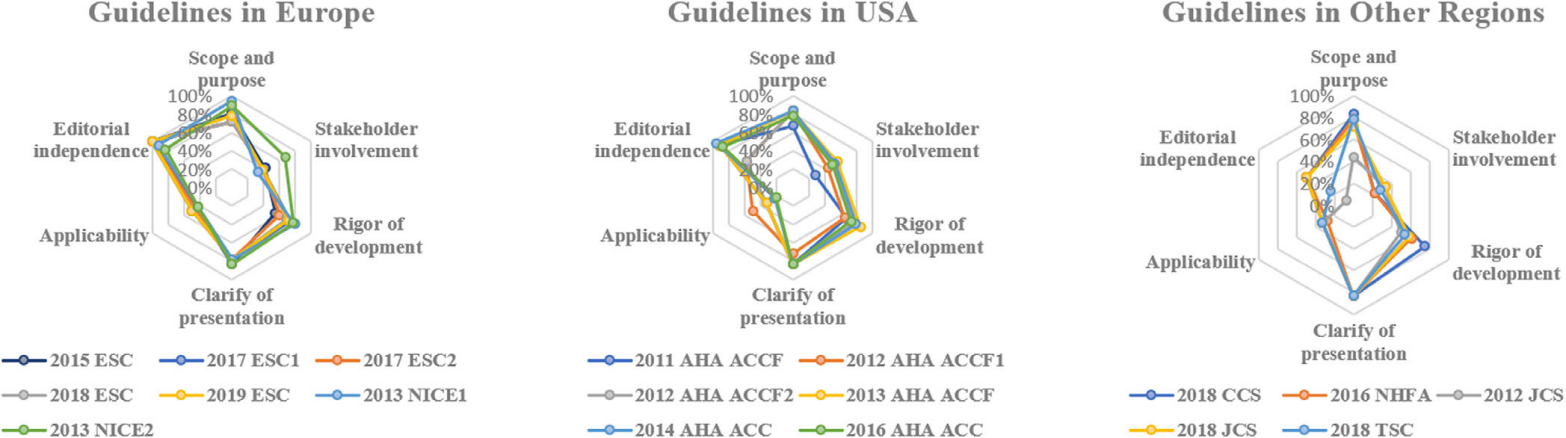
AGREE II domain scores of selection guidelines. ACC, American College of Cardiology; ACCF, American College of Cardiology Foundation; AHA, American Heart Association; CCS, Canadian Cardiovascular Society; ESC, European Society of Cardiology; JCS, Japanese Circulation Society; NHFA, National Heart Foundation of Australia; NICE, National Institute for Health and Care Excellence; TSC, Taiwan Society of Cardiology

The final scores of each domain for the included guidelines were shown in Figure 2 (specific scores could be found in Table S2). This illustrated the final scores for every guideline in each of the six domains. Higher domain scores were mapped towards the outer perimeter, and lower domain scores were plotted towards the center. The average AGREE II scores varied from 42% to 74%, with a median score of 66%. Of the 18 guidelines included, 10 (2019 ESC; 2018 ESC; 2017 ESC1; 2017 ESC2; 2016 AHA ACC; 2014 AHA ACC; 2013 AHA ACCF; 2011 AHA ACCF; 2013 NICE1; 2013 NICE2) were described as “strongly recommended” with average AGREE II scores ranged from 62% to 74%, while others were described as “recommended with modification.” No guideline was described as “not recommended for use in practice.” Reproducibility of the two reviewers' average AGREE II was good, with an intraclass correlation coefficient of 0.79.

For domain 1 (scope and purpose), most guidelines scored as high as around 80% and 2013 NICE1 even scored 94% for clearly describing their objective, health question and target population according to the principles of AGREE II.^5^ However, 2012 JCS did not define these items well and thus received a relatively low score of 47%.

In domain 2 (stakeholder involvement), most guidelines scored poorly (below 60%), for none of them had sought the views and preferences of the target population. Most guidelines did not explicitly show the detail of individuals in the development committee. In addition, 2011 AHA ACCF, 2016 NHFA, 2012 JCS, 2018 CCS and 2018 TSC did not clearly defined the target users of the guideline.

Domain 3 (rigor of development) showed a relatively good score among the guidelines with a wide range from 50% to 85%. Most guidelines provided the statement of literature searching for evidence. However, guidelines from ESC, JCS, NHFA/CSANZ, and TSC did not clearly mention the source of evidences, key words and the full search strategy. In addition, 2018 JCS, 2012 JCS, 2018 CCS, 2013 NICE2 and 2018 TSC did not mention the external review by experts before publication. Besides, 2015 ESC and 2018 TSC did not show the procedure for how the guideline was updated.

Domain 4 (clarity of presentation) is where these guidelines scored the best among six domains, with an average of 81%. Most of the recommendations in these guidelines were specific and unambiguous. Most guidelines clearly presented different options for management. Moreover, most of the key recommendations could be easily identified.

Domain 5 (applicability) got the poorest score averaging 38%, for these guidelines rarely described facilitators and barriers to the application. In addition, most of them did not provide advice or tools to help clinicians to put recommendations into practice, except for guidelines from European Society of Cardiology (ESC), which all provided educational tools and implementation programmers.^1^, ^12^, ^13^, ^14^, ^15^ Few clearly presented the monitoring and auditing criteria.

### 3.3 Appraisal of COI

For the reporting of COI, most guidelines got great score in domain 6 (editorial independence) for detailed provision of items according to the principle of AGREE II. However, 2012 JCS and 2018 TSC did not disclose any information about COI. For the complementary items from the RIGHT checklist, most guidelines disclosed these items well except for “the role of the funder(s) in guideline development, dissemination, and implementation,” as none of the guidelines presented it. However, no relationship between the average AGREE II scores and the proportion of panel members with an industry relationship was observed (Pearson's correlation *r* = −0.335; *P* = .205).

### 3.4 Recommendations for DAPT

Recommendations about DAPT in CAD were summarized in Table 2. Seventeen guidelines provided recommendations for patients treated with PCI. Eight guidelines mentioned recommendations for patients receiving medical therapy. Three guidelines were for patients with fibrinolytic therapy.

**Table 2:** Recommendations in guidelines on DAPT

					DAPT duration			
Treatment	Target populations	Guidelines	DAPT initial timing	P2Y12 inhibitor selection^a^	Stent type	Standard	High bleeding risk	High ischemic risk
PCI	SCAD	2019 ESC^12^	—	A + C/A + T/A + P^b^	DES/BMS/DCB	6 months	3 months	6‐30 months
		2018 ESC^13^	Before PCI^c^	A + C/A + T/A + P^b^	DES/BMS/DCB	6 months	3 months^d^	6‐30 months
					BRS	12 months	—	—
		2017 ESC1^14^	Before PCI^c^	A + C	DES/BMS/DCB	6 months	3 months^d^	6‐30 months
					BRS	12 months	—	—
		2016 AHA/ACC^18^	—	A + C	BMS	1 month^e^	—	—
					DES	6 months^e^	3 months	—
		2018 CCS^24^	—	A + C	BMS	6 months	1 month	12‐36 months
					DES		3 months	
		2012 JCS^26^	Before PCI	A + C	BMS/DES	1 month	—	—
	NSTE‐ACS	2018 ESC^13^	Before PCI	A + T/A + P > A + C^f^	—	12 months	6 months	>12 months
					BRS	12 months	—	—
		2017 ESC1^14^	Before PCI^g^	A + T/A + P > A + C^f^	DES/BMS/DCB	12 months^e^	6 months	>12 months
					BRS	12 months	—	—
		2015 ESC^15^	—	A + P/A + T > A + C	—	12 months	3‐6 months (DES)	>12 months
		2016 NHFA/CSANZ^25^	Before PCI	A + P/A + T > A + C	—	12 months	<12 months	>12 months
		2016 AHA/ACC^18^	—	A + T/A + P > A + C	BMS	12 months^e^	6 months	—
					DES	12 months^e^	—	—
		2014 AHA/ACC^19^	Before PCI	A + T/A + P > A + C	BMS/DES	≥12 months	—	—
		2012 AHA/ACCF1^21^	Before or at the time of PCI	A + C/A + T/A + P^h^	—	12 months	<12 months	—
		2011 AHA/ACCF^23^	—	A + T/A + P/A + C	BMS/DES	≥12 months	—	—
		2018 CCS^24^	—	A + T/A + P > A + C	—	12‐36 months	12 months	—
		2013 NICE2^17^	—	A + T/A + P/A + C	—	12 months	—	—
		2018 JCS^27^	—	A + C/A + P > A + T^i^	—	6‐12 months	<3 months	>12 months
		2018 TSC^28^	—	A + T/A + P > A + C^h^	—	12 months	—	>12 months
	STE‐ACS	2018 ESC^13^	Before PCI	A + T/A + P > A + C^f^	—	12 months	6 months	>12 months
					BRS	12 months		
		2017 ESC1^14^	Before PCI	A + T/A + P > A + C^f^	DES/BMS/DCB	12 months^e^	6 months	>12 months
					BRS	12 months	—	—
		2017 ESC2^1^	Before PCI	A + P/A + T > A + C^j^	—	12 months	6 months	12‐36 months
		2016 NHFA/CSANZ^25^	Before PCI	A + P/A + T > A + C	—	12 months	<12 months	>12 months
		2016 AHA/ACC^18^	—	A + T/A + P > A + C	BMS	12 months^e^	—	—‐
					DES	12 months^e^	6 months	—
		2013 AHA/ACCF^20^	Before or at the time of PCI	A + T/A + P/A + C	BMS	12 months	—	—
					DES	≥12 months		
		2011 AHA/ACCF^23^	—	A + T/A + P/A + C	BMS/DES	≥12 months	—	—
		2018 CCS^24^	—	A + T/A + P > A + C	—	12‐36 months	12 months	—
		2013 NICE1^16^	—	A + T/A + P/A + C	—	12 months	—	—
		2013 NICE2^17^	—	A + T/A + P/A + C	—	12 months	—	—
		2018 JCS^27^	Before PCI	A + C/A + P > A + T^i^	—	6‐12 months	<3 months	>12 months
Medical therapy	Stable CAD	2011 AHA/ACCF^23^	After SCAD	A + C	—	—	—	—
		2012 AHA/ACCF2^22^	After SCAD (certain high‐risk patients)	A + C	—	—	—	—
	ACS	2017 ESC 1^14^	After ACS	A + T > A + C^k^	—	12 months	≥1 month	12‐36 months
		2017 ESC 2^1^	After STEMI	—	—	12 months	—	—
		2016 AHA/ACC^18^	After ACS	A + T > A + C	—	12 months^e^	—	—
		2012 AHA/ACCF1^21^	After ACS	A + T/A + C	—	12 months	—	—
		2018 TSC^28^	After ACS	A + T > A + C	—	12 months	≥1 month	—
		2016 NHFA/CSANZ^25^	After ACS	A + T/A + C	—	12 months	<12 months	>12 months
Fibrinolytic therapy	STEMI	2016 AHA/ACC^18^	After fibrinolytic therapy	A + C	—	14 days to 12 months^e^	—	—
		2013 ACCF/AHA^20^	After fibrinolytic therapy	A + C	—	14 days to 12 months	—	—
		2017 ESC2^1^	After fibrinolytic therapy and subsequent PCI	A + C	—	12 months	—	

a A, aspirin; C, clopidogrel; P, prasugrel; T, ticagrelor.

b Prasugrel or ticagrelor may be considered in specific high‐risk situations of elective stent (eg, history of stent thrombosis or left main stenting) only.

c Pre‐treatment may be considered if the probability of PCI is high.

d 1 month if 3‐months DAPT poses safety concerns.

e The duration cloud be prolonged if the patients have tolerated DAPT without bleeding complication and are not at high bleeding risk.

f T is more preferred in patients with high ischemic risk and P is not recommended in patients with high bleeding risk.

g Pretreatment is recommended in patients in whom coronary anatomy is known and the decision to proceed to PCI is made.

h P is not recommended before PCI.

i T is considered in patients with prior myocardial infarction only.

j T is more preferred in patients with high ischemic risk.

k T is not recommended in patients with high bleeding risk.

### 3.5 Recommendations for DAPT in patients treated with PCI

Six guidelines suggested recommendations for patients with stable coronary artery disease (SCAD). For DAPT initial timing, 2012 JCS recommended pretreatment before PCI, 2018 ESC and 2017 ESC1 recommended pretreatment if the probability of PCI is high while 2019 ESC, 2016 AHA ACC and 2018 CCS did not mention it. For P_2_Y_12_ inhibitor, most guidelines suggested clopidogrel in addition to aspirin while 2018 ESC and 2019 ESC suggested ticagrelor and prasugrel are also indicated in high‐risk populations. For duration, most guidelines with relatively high AGREE II scores recommended 6 months standard treatment for patients with implantation of drug‐eluting stent (DES) while the low scores 2012 JCS suggested 1 month. For patients treated with bare‐mental stent (BMS), three guidelines (2019 ESC, 2018 ESC, 2017 ESC1) suggested 6 months while 2016 AHA ACC and 2012 JCS suggested 1 month as the standard duration. In addition, 2018 CCS did not clearly describe the type of stent for its recommended 6 months duration. What is more, only ESC guidelines provided suggestions for patients treated with bioresorbable vascular scaffolds (BRSs) or drug‐coated ball (DCB) (Table 2).

Twelve guidelines provided recommendations for patients with non‐ST‐elevation acute coronary syndrome (NSTE‐ACS). Four guidelines (2018 ESC, 2016 NHFA/CSANZA, 2015 AHA ACC, 2012 AHA ACCF1) recommended DAPT before PCI and 2017 ESC1 recommended pretreatment in patients in whom coronary anatomy is known and the decision to proceed to PCI is made. However, other seven guidelines did not mention about pretreatment. For P_2_Y_12_ inhibitor selection, most guidelines tended to favor ticagrelor or prasugrel over clopidogrel as an adjunct to aspirin. Most guidelines reached a consensus of 12 months as a standard treatment while 2018 JCS suggested 6 to 12 months. Half of the guidelines provide recommendations for patients with high bleeding risk. Among this, three guidelines (2018 ESC, 2017 ESC1, 2016 AHA ACC) suggested discontinuing at 6 months, two guidelines (2016 NHFA/CSANZ, 2012 AHA ACCF1) suggested discontinuing before 12 months, one guideline (2015 ESC) suggested 3 to 6 months, one guideline (2018 JCS) suggested 3 months while 2018 CCS suggested discontinuing at 12 months. For patients with high ischemic risk, six guidelines (2018 ESC, 2017 ESC1, 2015 ESC, 2016 NHFA/CSANZ, 2018 JCS, 2018 TSC) considered continuing for longer than 12 months (Table 2).

Eleven guidelines suggested recommendations for patients with ST‐elevation ACS. For DAPT initial timing, half of the guidelines (2018 ESC, 2017 ESC1, 2017 ESC2, 2016 NHFA/CSANZ, 2013 AHA ACCF) recommended pretreatment before PCI while other guidelines (2016 AHA ACC, 2011 AHA ACCF, 2018 CCS, 2013 NICE1, 2013 NICE2) did not mention about. In addition, recommendations for P_2_Y_12_ inhibitor selection or DAPT duration were similar to that for NSTE‐ACS (Table 2).

### 3.6 Recommendations for DAPT in patients treated with medical therapy

Only two guidelines (2011 AHA ACCF, 2012 AHA ACCF2) mentioned DAPT in patients with medically managed SCAD. Both of them recommended aspirin plus clopidogrel without clear duration. Six guidelines recommended DAPT in patients with ACS receiving medical therapy. Most guidelines recommended ticagrelor or clopidogrel plus aspirin for 12 months as a standard treatment. Three guidelines provided suggestions for duration adjustment on grounds of high ischemic or bleeding risk, which basically reached a consensus.

### 3.7 Recommendations for DAPT in patients treated with fibrinolytic therapy

Three guidelines (2016 AHA ACC, 2013 ACCF/AHA, 2017 ESC2) recommended DAPT in patients with STEMI treated with fibrinolytic therapy. All of them suggested clopidogrel in addition to aspirin after fibrinolytic therapy. 2016 AHA/ACC and 2013 AHA/ACCF suggested the duration should be continued for at least 14 days and up to 12 months. 2017 ESC2 recommended up to 12 months in patients undergoing fibrinolysis and subsequent PCI.

## 4 DISCUSSION

In general, we identified 18 guidelines with recommendations on DAPT for patients with CAD. To the best of our knowledge, it was the first comprehensive evaluation of the international DAPT guidelines. Based on the rigorous systematic assessment recommended by the AGREE II appraisal instrument, we found the general quality of these guidelines to be relatively high, though minor deficiencies still existed. And for the recommendations, there were still inconsistencies primarily with regards to DAPT duration.

Several recently published articles have assessed the quality of clinical guidelines using AGREE II tool, including various topics from different specialties.^6^, ^29^, ^30^ Low scoring domains in these studies were found mostly in domain 2 “stakeholder involvement,” domain 3 “rigor of development” and domain 5 “applicability,” which reflected the widespread problem of the existing guidelines in different specialties. In our study, it is somewhat reassuring to note that, most of these DAPT‐relevant guidelines, especially more recent guidelines of ESC, AHA/ACC and NICE got relatively good score in domain “rigor of development.” This showed that most DAPT guidelines were strictly followed by evidence‐based principles when they were developed. It was significant as it ensured the DAPT recommendations in these guidelines were based on evidence and were credible. However, similar to other studies, we found most DAPT guidelines got a low score in domain “stakeholder involvement” and “applicability.” In contrast to the rigor of the guidelines, most guidelines did not attach importance to the representativeness of the guidelines' stakeholder and the application of the recommendations. In addition, none of the guidelines presented the views and preferences of the target population, which would limit the judicial use of the guidelines in various scenarios. Improving this item would help make the clinical guidelines more accessible to a wider audience.^31^ Also, many guidelines failed to present the information regarding the individuals of the development group well. The incomplete professional groups may also impact the applicability of the guidelines and lead to the lower scores in the “applicability” domain. In this domain, most guidelines did not pay attention to the facilitators or barriers to its application. For example, only a few guidelines provided DAPT strategies for patients with fibrinolytic therapy. Although PCI has become the standard of care for ST‐segment elevation myocardial infarction, only a few hospitals can provide on‐site, 24‐hour access to primary PCI in the United States,^32^ let alone in developing countries. Similarly, failure to consider potential resources for the recommendations, such as the cost of using the new drugs for DAPT, may also affect the application of these guidelines.

The reporting of COI is one of the important portions in guideline development, which help minimizing the risk of bias associated with COI and increases overall credibility.^11^ In DAPT‐relevant guidelines, COI may influence the recommendations for P_2_Y_12_ inhibitor selection and DAPT duration. AGREE II makes an evaluation on COI with two items in domain 6 particularly. We critically appraised of this domain and the general scores were good except for 2018 TSC and 2012 JCS. However, in our opinions, the items were still not detailed enough. To compensate for this, Chen et al developed a tool—the RIGHT checklist, which provides the complementary items to appraise the quality of the reporting of COI.^10^, ^11^ We systematically evaluated these items and found that the majority of these guidelines agreed with the items except for: “the role of the funder(s) in guideline development, dissemination, and implementation.” Addressing this item would well increase the credibility of the DAPT guidelines. As mentioned above, guidelines 2018 TSC and 2012 JCS provide little information about COI. Completing the reporting will well improve the quality of these guidelines. Besides, we also examined the correlation between the proportion of guideline panel members who reported relationships with industry and the AGREE II score, but no relationship was identified.

It is generally believed that appropriate use of high‐quality guidelines can improve patient outcomes.^3^, ^31^ In Komajda et al's study,^33^ physician's guideline adherence is associated with better prognosis among outpatients, which emphasizes the importance of guideline‐directed medical therapy. As a result, it is of great significance to improve the defects found in this assessment.

With respect to the recommendations, most guidelines reached general consensus, though discrepancies still existed, particularly in DAPT duration. For recommendations on SCAD patients managed with BMS implantation, 2016 AHA ACC and 2012 JCS came to an agreement on 1 month while 2019 ESC, 2018 ESC, 2017 ESC1 recommended 6 months. We critically reviewed the evidences presented by the guidelines.^34^, ^35^, ^36^, ^37^ Although they provided high quality evidences for the appropriate use of DAPT for 1 month or 6 months separately, no dedicated studies comparing these two durations for SCAD patients undergoing PCI were present. As a result, more evidences are needed. In addition, 2012 JCS (with relatively low scores) did not provided any evidence so more information should be presented. For patients with implantation of DES, four strongly recommended guidelines 2019 ESC, 2018 ESC, 2017 ESC1, and 2016 AHA ACC unanimously recommended 6 months and presented high quality evidence. On the contrary, 2012 JCS recommended 1 month without citing any evidence, which may be unreliable. What is more, 2018 CCS should represent clearly in terms of the stent type for the subsequent strategy may be different.

Although there are differences between NSTE‐ACS and STE‐ACS in pathophysiology, the recommendations on DAPT are similar, which may due to the study design of their relevant evidences. For NSTE‐ACS or STE‐ACS patients treated with PCI, the recommendations for DAPT initial timing are controversial. Nearly half of guidelines suggested pretreatment before PCI while half of guidelines did not mention. However, in the explanatory section, guidelines all expressed that, due to the lacking of evidence, whether patients undergoing PCI should be pretreated is stilled debated.^13^, ^14^, ^19^, ^21^, ^25^ As a result, more evidences are needed to generate the strategy. As for DAPT duration, most guidelines came to an agreement of 12 months as the standard duration. Tendency could be seen from Table 2 that recently published guidelines tended to provide individual duration adjustment for patients with different bleeding or ischemic risk, although there were still some inconsistencies.

In the last 23 years, various lengths of appropriate duration were proposed.^38^ Although these studies provided significant amount of data, the results of the main studies were apparently inconsistent because of the differences in study designs, type of coronary devices, and targeted populations.^2^ As more and more evidence accumulated, major controversy revolved around DAPT prolongation or shortening of the standard duration.^14^, ^38^ Premature discontinuation of DAPT before standard duration may lead to stent thrombotic events,^39^, ^40^, ^41^ while shortened duration is associated with lower bleeding rate and non‐inferiority cloud be found in several studies.^42^, ^43^, ^44^ On the contrary, extension of DAPT provides protection against the risk of late sent thrombosis. However, the accompanied high bleeding risk would also affect morbidity and mortality. As a result, decision about DAPT duration should be tailor‐made, balancing the individual patient's risk of ischemia and bleeding. In our study, we found that 2017 ESC1 and 2016 AHA, the first guideline focused in DAPT of each organization, both provided risk stratification tools (PRECISE‐DAPT score and DAPT score) for ischemia and bleeding risks. In our opinion, it was a great advance in the development of DAPT, which help clinicians to evaluate the trade‐off between ischemic vs bleeding risk and choose the best DAPT strategy for the individual patients. Clinicians usually pay more attention to the practical application when they read the guidelines. Applying these tools would make it easier for clinicians to use the DAPT guidelines without many difficulties, which would improve the application of the guidelines and the score of domain 5. It would be meaningful for other organization to pay more attention to the risk stratification tools when updating their guidelines. However, none of the risk prediction models in these guidelines have been prospectively tested by RCTs and consequently their value in improving patient outcomes remain uncertain.^14^ The question of the optimal duration for DAPT warrants further investigation.

What is more, for either SCAD or ACS patients, only two recent guidelines 2018 ESC and 2017 ESC1 provided recommendations on DAPT in patients treated with BRS or DCB. Evidence for BRS came from one randomized controlled trial (RCT)^45^ published later than most guidelines, which investigated the treatment of patients with BRS and the DAPT duration provided in this study was 12 months. Evidence for DCB came from three RCTs,^46^, ^47^, ^48^ which explored the effect of DCB. However, the duration of DAPT in these studies was inconsistent. It would be meaningful for other organizations to update their recommendations on DAPT for patients who receive BRS or DCB. And more evidences are needed to refine these recommendations.

There are several possible limitations in our study. First, only guidelines published in English were included. Hence, many meaningful guidelines from non‐English regions were not included in our study. Besides, one of the guidelines in our study from Japan, the 2012 JCS, was only a digest version published in English and did not provide the detailed information of its own development. This may account for the low AGREE II scores of it. In addition, the only three guidelines from Asia in our study could not represent other Asian countries. Second, the AGREE II tool considers the reported information only, whereas the real quality of the development of the guidelines may or may not be provided. As a result, a guideline developed well may also get a low score. Third, the AGREE II tool provides the overall scores of the developmental process of guidelines. However, a poorly developed guideline may also provide a reliable recommendation. A recommendation of low quality may also come from a high score guideline. Fourth, considering the simplicity and readability of the recommendation table, we did not include the level of evidence for each recommendation in it, though it is important in the comparison of recommendations from different guidelines. Finally, it is difficult to comprehensively evaluate the potential conflict of interest simply by reviewing the guidelines.

## 5 CONCLUSION

Quality of guidelines for DAPT in CAD was relatively high, though defects existed in “Applicability” and “Stakeholder Involvement.” As these guidelines developed, DAPT recommendations gradually converged on a consensus. Clinical decision should be made on an individual basis.

## CONFLICT OF INTEREST

The authors declare no potential conflict of interests.

## AUTHOR CONTRIBUTIONS

Zhimin Du, Xinxue Liao and Shaozhao Zhang devised the study. Shaozhao Zhang, Huimin Zhou and Xiuting Sun extracted, analyzed and interpreted the data. Shaozhao Zhang, Huimin Zhou, Xiuting Sun and Xiaodong Zhuang wrote the first draft. All authors contributed to subsequent versions and approved the final article. Zhimin Du and Xinxue Liao are the corresponding authors. All authors read and approved the final manuscript.

## Supporting information


**Table S1**. Websites of guideline organizations and professional societies.

**Table S2**. Overall AGREE II domain scores.
