# Supplementary material for: Critical appraisal of guidelines for coronary artery disease on dual antiplatelet therapy: More consensus than controversies
Source: Clin Cardiol. 2019 Oct 14;42(12):1170–80. doi: 10.1002/clc.23275 (PMC6906997; doi:10.1002/clc.23275)
Supplement: Supplementary file 1 — Table S1. Websites of guideline organizations and professional societies. [file CLC-42-1170-s001.docx]

**Websites of guideline organizations and professional societies.**

| ESC | https://www.escardio.org/Guidelines |
| --- | --- |
| ACC | https://www.acc.org/guidelines#doctype=Guidelines |
| NICE | https://www.nice.org.uk/guidance |
| CCS | http://www.ccs.ca/en/guidelines |
| NHFA | https://www.heartfoundation.org.au/for-professionals/clinical-information |
| JCS | http://www.j-circ.or.jp/guideline/ |
