# Supplementary material for: Critical appraisal of guidelines for coronary artery disease on dual antiplatelet therapy: More consensus than controversies
Source: Clin Cardiol. 2019 Oct 14;42(12):1170–80. doi: 10.1002/clc.23275 (PMC6906997; doi:10.1002/clc.23275)
Supplement: Supplementary file 2 — Table S2. Overall AGREE II domain scores. [file CLC-42-1170-s002.docx]

**Table S1.** **Overall AGREE II domain scores.**

|  | Domain 1 | Domain 2 | Domain3 | Domain4 | Domain5 | Domain6 |
| --- | --- | --- | --- | --- | --- | --- |
| 2019 ESC | **78%** | **39%** | **71%** | **78%** | **50%** | **100%** |
| 2018 ESC | **72%** | **39%** | **69%** | **78%** | **50%** | **100%** |
| 2017 ESC1 | **72%** | **39%** | **73%** | **78%** | **50%** | **100%** |
| 2017 ESC2 | **72%** | **39%** | **60%** | **78%** | **46%** | **100%** |
| 2015 ESC | **81%** | **42%** | **55%** | **83%** | **44%** | **100%** |
| 2013 NICE1 | **94%** | **33%** | **79%** | **78%** | **42%** | **92%** |
| 2013 NICE2 | **89%** | **67%** | **77%** | **83%** | **42%** | **83%** |
| 2016 AHA ACC | **78%** | **50%** | **73%** | **83%** | **21%** | **88%** |
| 2014 AHA ACC | **83%** | **53%** | **79%** | **83%** | **23%** | **96%** |
| 2013 AHA ACCF | **83%** | **56%** | **85%** | **83%** | **33%** | **92%** |
| 2012 AHA ACCF1 | **83%** | **44%** | **65%** | **72%** | **50%** | **58%** |
| 2012 AHA ACCF2 | **83%** | **50%** | **77%** | **83%** | **33%** | **58%** |
| 2011 AHA ACCF | **67%** | **28%** | **67%** | **83%** | **33%** | **92%** |
| 2018 CCS | **83%** | **22%** | **75%** | **83%** | **33%** | **50%** |
| 2016 NHFA/CSANZ | **78%** | **22%** | **61%** | **83%** | **28%** | **50%** |
| 2012 JCS | **44%** | **33%** | **50%** | **83%** | **33%** | **8%** |
| 2018 JCS | **72%** | **33%** | **50%** | **83%** | **33%** | **54%** |
| 2018 TSC | **78%** | **28%** | **54%** | **83%** | **33%** | **25%** |
